# Imaging Inflammation in Patients and Animals: Focus on PET Imaging the Vulnerable Plaque

**DOI:** 10.3390/cells10102573

**Published:** 2021-09-28

**Authors:** Benjamin Bartlett, Herbert P. Ludewick, Silvia Lee, Shipra Verma, Roslyn J. Francis, Girish Dwivedi

**Affiliations:** 1 Department of Advanced Clinical and Translational Cardiovascular Imaging, Harry Perkins Institute of Medical Research, Murdoch, WA 6150, Australia; benjamin.bartlett@research.uwa.edu.au (B.B.); herbert.ludewick@uwa.edu.au (H.P.L.); silvia.lee@uwa.edu.au (S.L.); 2 School of Medicine, University of Western Australia, Perth, WA 6009, Australia; roslyn.francis@uwa.edu.au; 3 Heart and Lung Research Institute, Harry Perkins Institute of Medical Research, Murdoch, WA 6150, Australia; 4 Department of Microbiology, Pathwest Laboratory Medicine, Perth, WA 6909, Australia; 5 Department of Nuclear Medicine, PET CT and Radionuclide Therapy, Fiona Stanley Hospital, Murdoch, WA 6150, Australia; shipra.verma@health.wa.gov.au; 6 Department of Geriatric Medicine, Fiona Stanley Hospital, Murdoch, WA 6150, Australia; 7 Department of Nuclear Medicine, Sir Charles Gairdner Hospital, Nedlands, WA 6009, Australia; 8 Department of Cardiology, Fiona Stanley Hospital, Murdoch, WA 6150, Australia

**Keywords:** PET imaging, atherosclerosis, vulnerable plaque, inflammation

## Abstract

Acute coronary syndrome (ACS) describes a range of conditions associated with the rupture of high-risk or vulnerable plaque. Vulnerable atherosclerotic plaque is associated with many changes in its microenvironment which could potentially cause rapid plaque progression. Present-day PET imaging presents a plethora of radiopharmaceuticals designed to image different characteristics throughout plaque progression. Improved knowledge of atherosclerotic disease pathways has facilitated a growing number of pathophysiological targets for more innovative radiotracer design aimed at identifying at-risk vulnerable plaque and earlier intervention opportunity. This paper reviews the efficacy of PET imaging radiotracers ^18^F-FDG, ^18^F-NaF, ^68^Ga-DOTATATE, ^64^Cu-DOTATATE and ^68^Ga-pentixafor in plaque characterisation and risk assessment, as well as the translational potential of novel radiotracers in animal studies. Finally, we discuss our murine PET imaging experience and the challenges encountered.

## 1. Introduction

The goal of non-invasive imaging of atherosclerotic plaques is to enable better prediction of cardiovascular (CV) events by improving assessment of asymptomatic, at-risk plaque. Currently, the two main screening techniques in place are invasive coronary angiography and coronary computed tomography (CT) angiography (CCTA). The gold standard for identifying coronary artery stenosis is invasive coronary angiography, which is an invasive procedure with attending risks [1]. CCTA utilises an imaging contrast agent in combination with x-rays and computer technology to visualise both hard and soft plaques non-invasively. These imaging techniques are relatively quick and accurate, but they are unable to provide molecular and cellular-level details provided by positron emission tomography (PET) imaging [2].

**Figure 1:**
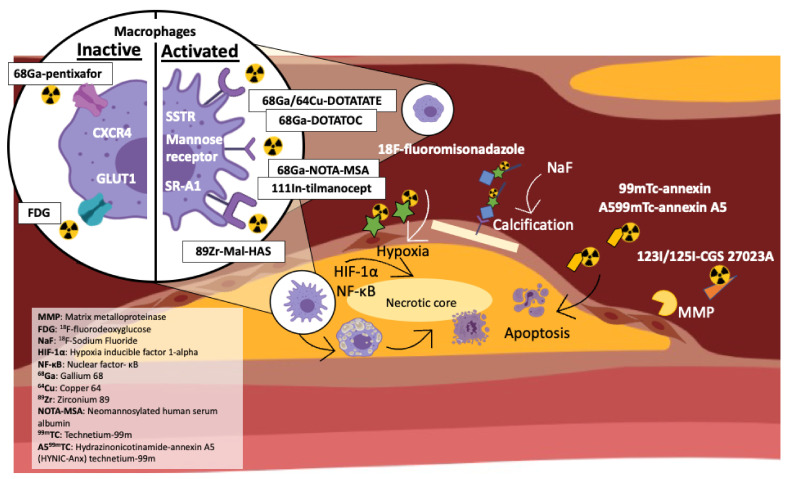
Schematic representation of pathophysiological pathways and the radiotracers that can image them. FDG is taken up by high metabolically active cells (i.e., inflammatory cells including macrophages). NaF targets micro- and macro-calcification and is typically used to identify vulnerable plaque. 123l/125l-CGS 27023A targets MMP plaque activity, associated with plaque vulnerability. ^18^F-fluoromisonadazole targets hypoxia. 99mTc-annexin and A599mTc-annexin A5 target cell apoptosis. 68Ga/64Cu-Dotatate, 68Ga-DOTATOC, 68Ga-NOTA-MSA, 111In-tilmanocept and 89Zr-Mal-HAS target activated macrophages. 68Ga-pentixafor targets macrophages and T-cells.

PET imaging has high sensitivity for the detection of sparse targets in the nanomolar range with low tracer doses [3]. Although it is limited to anatomical structure, in combination with CT or magnetic resonance imaging (MRI), it results in effective molecular and structural imaging. PET imaging in cardiovascular disease (CVD) offers valuable insight into identifying atherosclerotic plaque activity, myocardial perfusion and viability, and measuring the extent of cardiac innervation in heart failure. It is proving to be an invaluable imaging modality for assessing plaque rupture risk due to the ability of radiotracers to identify molecular changes symbolic of vulnerable plaques [4]. The range of radiotracers available enables PET imaging to visualise different biological processes and molecular pathways throughout atherogenesis and identify plaques at risk of becoming asymptomatic [5] (Figure 1).

Specific radiotracers in PET imaging have been extensively utilised in providing insight into patients with acute coronary syndrome (ACS). ACS represents an end-stage of atherosclerotic plaque development and rupture. Present-day PET imaging presents the option of choosing different radiopharmaceuticals to image different plaque characteristics throughout this process. The focus of this review is to discuss the utility of PET imaging in providing insight into assessing vulnerable plaque, its use in animal models, and its future direction.

## 2. Lipid Accumulation & Inflammation in Plaque Development

Atherosclerosis is initiated by the deposition and accumulation of lipids and fibrous elements in the arterial wall [6]. Plaque development and progression is further initiated and largely driven by an innate immune response [7]. Low density lipoproteins (LDLs) are oxidised (oxLDL), promoting monocyte/macrophage recruitment and inducing an immune response [8]. Phagocytosis of oxLDL by innate immune cells, primarily macrophages, results in the formation foam cells and fatty streaks. The accumulation of lipids and leukocyte infiltration contributes to the formation of a necrotic core, tissue remodelling, and the development of a collagen-rich fibrous cap established by vascular smooth muscle cells [6,9].

## 3. ^18^F-Fluorodeoxyglucose (FDG) -PET Detects Plaque Development and Inflammatory Cell Infiltrate

FDG is a glucose analogue and the most-validated radiotracer for imaging high metabolically active inflammatory cells (e.g., macrophages) and tissues (e.g., atherosclerotic plaques) in animal models and humans [10]. The results have proven to be reproducible and modifiable via interventions that are anti-inflammatory [11]. FDG-PET imaging may mirror inflammatory activity in atherosclerosis due to the consumption of large amounts of glucose by inflammatory cells compared to other plaque cells.

The interpretation of the uptake of glucose by inflammatory cells and non-specific uptake of cells in the arterial wall could prove challenging. The different subtypes of inflammatory macrophages have divergent roles in plaque development and progression. M1 macrophages are pro-inflammatory and more glycolytically active than M2 anti-inflammatory cells [12]. Another concerning factor that can also affect imaging results and outcome is the non-specific uptake by highly glycolytic cells in the arterial wall [13]. However, there are inconsistent reports in this area [14]. Tavakoli and colleagues hypothesized that differential regulation of macrophage metabolism by macrophage colony-stimulating factor (M-CSF; inflammatory resolving) and granulocyte-M-CSF (GM-CSF; proinflammatory) may contribute to the inconsistency of FDG vessel wall inflammation [14]. The metabolic profiles generated comparable levels of glucose uptake in cultured macrophages and murine atherosclerotic plaques. These findings suggest that although FDG uptake is an indicator of vascular macrophage burden and numbers, it may not necessarily differentiate morphologically unstable from stable plaque, or identify those at risk of rupture and symptomatic atherothrombosis [15]. Moreover, there is a wide range of vascular diseases in which macrophages and inflammation play an important role in the absence of atherosclerosis [15]. These include large artery inflammatory vascular diseases such as Takayasu arteritis, chemotherapy- or radiation-induced vascular inflammation, or foreign body reaction such as synthetic arterial graft. Due to the low sensitivity and non-specific nature of FDG uptake, caution is needed when interpreting vascular FDG uptake as a sole indicator of inflammatory atherosclerosis. What is critically needed for FDG-PET to become a major imaging modality for atherosclerosis is a prospective, event-driven investigation that links plaque FDG uptake to patient outcome [15].

Experimental studies of FDG-PET in atherosclerosis have shown that distribution of FDG within atherosclerotic plaques occurs predominantly in macrophages, and FDG uptake correlates with plaque inflammation in clinical imaging [16]. However, a consensus regarding the most appropriate FDG thresholds for defining plaque vulnerability is lacking, primarily because healthy patients, presumably without pathological arterial inflammation, have not, to our knowledge, been systematically imaged [17]. Arterial FDG uptake was recently assessed in healthy control patients, those with risk factors, and patients with CVD to derive both uptake thresholds in each patient group and the reproducibility of the measures. Although the measured FDG metrics were reproducible and significantly different between patients who were healthy and who had disease, there was data overlap between patient categories, making FDG a non-specific signal for plaque inflammation and limiting its generalizability [17,18].

In addition, uptake of FDG in the heart, an organ of high metabolic activity, can present challenges in assessing inflammation [19,20]. This becomes of concern in the coronary arteries, where spillover from the physiologic activity of the heart obscures detection and accurate quantification of FDG uptake and plaque inflammation [19].

## 4. ^18^F-Sodium Fluoride (^18^F-NaF) PET Predicts Plaque Calcification

^18^F-NaF is another radiotracer of interest. As the myocardium does not take up ^18^F-NaF, uptake can be easily detected in coronary plaque without confounding uptake from the myocardium (as observed with FDG). This practical consideration simplifies the application of ^18^F-NaF-PET imaging in patients [21].

A characteristic feature of plaque development involves microcalcification (0.5–15 μm), a process dependent on inflammation resulting in the development of larger sheet-like deposits (> 3 mm) [22,23]. A number of imaging modalities propose that spotty microcalcification is a predictor of unstable plaque, whereas more extensive calcification is more resistant to changes in volume and is associated with stable plaques [22]. Serial intravascular ultrasound studies report that spotty calcification is associated with greater plaque progression and volume compared to non-calcified plaques [24,25]. Additionally, this tracer could also be useful in the disease stratification of patients with stable plaque before an adverse event, and further characterise risk in patients with vulnerable plaque detected by CCTA [26]. Advanced atherosclerosis is associated with the phenotypic conversion of vascular myofibroblasts into osteoblastic cells, promoting calcification [27]. On a distensible surface such as the vascular endothelium, a mismatch can occur, making it more prone to rupture at the tissue–calcium interface/conjunction [28]. ^18^F-NaF-PET imaging targets this calcification process, and the uptake of NaF can assess plaque stability through measuring calcification. Arterial calcification is an independent predictor of an adverse CV event. It is now widely accepted that calcification associates with plaque progression and vulnerability. Microcalcifications provide further stimulus for inflammatory response and thus perpetuate the inflammatory cycle, leading to plaque instability [29]. Within the lesion, macrophages can alter their phenotype to resolve the inflammation and induce regression or stabilization of the plaque; this is often observed in the macrocalcification process [30]. In later stages of the healing process, cells support the development of the extracellular matrix and facilitate plaque calcification, leading to a more stable plaque phenotype [31].

A recent review stated that ^18^F-NaF-PET correlates with CV risk factors, and ^18^F-NaF uptake appears to be a good measure of the body’s atherosclerotic burden, potentially suited for assessment of anti-atherosclerosis therapy [26]. Studies also observed that age and CV risk were associated with prominent increases in vascular calcification in the abdominal aorta, providing more evidence suggesting that ^18^F-NaF may serve as a potential biomarker for vulnerability and CV risk [32,33,34]. This is in line with a study showing significantly higher ^18^F-NaF uptake in patients with high CV risk factors and thoracic fat volume [35].

A prospective study of 80 patients utilising ^18^F-NaF-PET imaging was able to successfully identify vulnerable coronary lesions in 93% of patients with myocardial infarction [21]. Increased uptake was also observed in 45% of patients with stable coronary artery disease (CAD) (patients referred for invasive coronary angiography). Regions of ^18^F-NaF uptake in the patients correlated with intravascular ultrasound findings of microcalcification, a necrotic core, and positive remodelling.

Myung et al. demonstrated that coronary plaques with high-risk characteristics on intravascular imaging (ultrasound and optical coherence tomography) had higher ^18^F-NaF uptake compared to those without those characteristics [36]. Moreover, Kitagawa made the observation that (1) high plaque ^18^F-NaF uptake correlated positively with coronary calcium score per patient, (2) patients with a history of myocardial infarction or unstable angina have a higher coronary artery ^18^F-NaF uptake, (3) increased ^18^F-NaF uptake in coronary atherosclerosis is independently correlated with partially calcified plaque components, and (4) coronary plaques with high-risk characteristics present with higher ^18^F-NaF uptake on PET than those without [37].

Several studies have investigated the utility of ^18^F-NaF-PET in predicting coronary events. High ^18^F-NaF uptake is predictive of a coronary event within the next 2 years, correlating with advanced coronary calcification and presenting as a high-risk plaque on CCTA. The results support implementation of CCTA and ^18^F-NaF-PET for non-invasive identification of high-risk CAD [38]. More recently, Kwiecinski and colleagues assessed whether ^18^F-NaF-PET could help predict myocardial infarction and provide any additional prognostic information to current methods of risk stratification [39]. Measuring coronary microcalcification activity as total coronary ^18^F-NaF uptake was found to be better for predicting fatal or nonfatal myocardial infarction compared to coronary calcium scoring, the modified Duke CAD index, and the Reduction of Atherothrombosis for Continued Health (REACH) and Secondary Manifestations of Arterial Disease (SMART) risk scores.

When utilising ^18^F-NaF-PET imaging to quantify coronary artery plaque burden, the challenge of quantification due to low target to background ratios, partial volume effects and motion must also be considered [13]. However, altogether, a large number of studies highlight the potential of ^18^F-NaF-PET imaging as an innovative approach to monitoring the disease progression and vulnerable plaques in atherosclerosis.

## 5. Somatostatin 2 Receptor (SSTR) Imaging

### 5.1. Gallium DOTATATE (^68^Ga-DOTATATE) and Plaque Identification

^68^Ga-DOTATATE has become an attractive option in PET imaging due to its high specificity for the G-coupled receptor somatostatin receptor subtype-2 (SSTR2), which is up-regulated in activated macrophages [40,41].

The low physiological expression of SSTR2 by the myocardium suggests that this tracer may be advantageous for imaging disease in the coronary arteries [42]. Several studies have validated the expression of SSTR2 in preclinical murine models (*ApoE-/-* mouse model) at the tissue level [43,44]. In humans, the efficacy of ^68^Ga-DOTATATE to ^18^F-FDG evaluated in 42 patients with atherosclerosis was found to offer superior coronary imaging. ^68^Ga-DOTATATE demonstrated excellent macrophage specificity and better discriminative power to identify high-risk versus low-risk coronary lesions compared to ^18^F-FDG [45]. Furthermore, low levels of SSTR2 were detected in unstimulated macrophages and alternatively activated M2 subtypes, but not in other cell types (monocytes, T or B lymphocytes, natural killer cells, platelets, neutrophils, and endothelial cells). The study also observed specific ^68^Ga-dotatate binding to SSTR2 within areas of CD68^+^ macrophage-rich carotid plaque regions with a strong correlation of carotid SSTR mRNA and in vivo ^68^Ga-DOTATATE activity [45]. The study presents strong evidence of ^68^Ga-DOTATATE as a valid investigation tool for unstable plaque identification.

In contrast, a prospective study evaluating ^68^Ga-DOTATATE uptake in carotid plaque of patients with recent carotid events found no difference between recently symptomatic carotid plaques vs. contralateral plaques [46]. Despite the presence of CD68^+^ macrophages in vitro, SSTR2 expression was not detected in excised plaques.

The majority of studies have demonstrated potential use for ^68^Ga-DOTATATE in preclinical and early human studies however, the radiotracer still warrants further characterisation to verify its role in vulnerable plaque risk stratification [46,47].

### 5.2. Dota Derived Somatostatin Analogue ^68^Ga-DOTATOC

^68^Ga-DOTATOC shares a similar SST binding profile to ^68^Ga-DOTATATE, but its binding affinity to SSTR is 10-fold lower [48,49]. The feasibility of ^68^Ga-DOTATOC-PET for assessing vulnerable plaque in the thoracic aorta was investigated, and the study found uptake correlated with CV risk factors [50]. Furthermore, the study also assessed quantification methods, comparing the difference in uptake quantification between multi-sample region of interest and single volume of interest methods to assess the efficacy of measurement indexes in terms of CV risk factors. The results exhibited high correlation between the two methods of assessing uptake, but commented that the aortic arch would challenge reproducible measurements due to its complex geometry. The study also notes that the uptake of ^68^Ga-DOTATOC significantly correlates with the Framingham risk score, a measure of CV risk, corresponding with earlier studies suggesting a role as a predictor of CV events and as a biomarker for vulnerable plaque assessment [45,49,50,51,52,53].

The significant correlation of ^68^Ga-DOTATOC uptake with CV risk factors suggests its use as a potential predictor for CV events and a biomarker for the assessment of vulnerable plaque. Further studies exploring the clinical efficacy and relevance of ^68^Ga-DOTATOC are warranted to validate the value of ^68^Ga-DOTATOC PET/CT in atherosclerosis and its correlation with CV risk and events and PET indexes [50].

### 5.3. ^64^Cu-DOTATATE

^64^Cu-DOTATATE, like the other DOTATATE and DOTATOC variants, targets the SSTR expressed on activated macrophages which accumulate in active inflammatory lesions. A study in humans found that vascular uptake of ^64^Cu-DOTATATE was higher than ^68^Ga-DOTATOC, suggesting a potential role of ^64^Cu-DOTATATE in the assessment of atherosclerosis [49].

In a study of 10 patients who underwent carotid endarterectomy, uptake of ^64^Cu-DOTATATE correlated with gene expression of CD163, a surrogate marker of alternatively activated macrophages within atherosclerotic plaques [52]. This finding in particular is of interest due to the role of CD163 macrophages in haemorrhagic zones [54]. Interestingly, there was no correlation between plaque burden and ^64^Cu-DOTATATE uptake [52]. This observation could potentially help improve non-invasive identification and characterization of vulnerable plaques [52].

Markers of plaque vulnerability (including cathepsin K, matrix metalloproteinase-9 (MMP-9), and IL-18), previously found to be associated with FDG uptake, did not correlate with ^64^Cu-DOTATATE uptake [52]. This clearly highlights the difference in targeting between these radiotracers.

## 6. Chemokine Imaging

The C-X-C motif chemokine receptor 4 (CXCR4) is expressed on the surface of various cell types involved in atherosclerosis, including macrophages and T-cells [55]. Its role and its endogenous ligand C-X-C motif chemokine 12 (CXCL12) in atherosclerosis is yet to be fully elucidated [56]. CXCR4 and CXCL12 fulfil important roles in progenitor and immune cell trafficking; however, there are conflicting reports of atherogenic and atheroprotective effects [57]. The conflict is partly due to the identification of migration inhibitory factor as an alternative ligand for CXCR4 [58]. Evidence demonstrates that CXCR4 activation by CXCL12 exerts a stabilizing effect on atherosclerotic lesions, whereas migration inhibitory factor acts as a major pro-inflammatory player.

A study of human carotid plaques found CXCR4 expression was elevated in both stable and unstable atherosclerotic plaques, with the highest receptor expression found in macrophage-derived foam cells and macrophages [59]. Similarly, in rabbits, ^125^I-pentixafor accumulated in inflamed plaques, which was verified histologically by the detection of macrophages and CXCR4 in plaques of the abdominal aorta and carotid artery [60]. Together, these findings present a complex system of CXCR4-expressing cell types that, depending on the activating ligand, may have athero-protective or atherogenic effects.

### ^68^Ga-Pentixafor

A recent study evaluated the performance of ^68^Ga-pentixafor and ^18^F-FDG for the detection of arterial wall inflammation and calcification in lesions [55]. The retrospective study of 92 patients found that ^68^Ga-pentixafor identified a greater number of atherosclerotic lesions with higher uptake compared to ^18^F-FDG. ^68^Ga-pentixafor detected sites that were overlooked on FDG-PET, suggesting that the pentixafor uptake originates from cell types beyond inflammation [61]. CXCR4-expressing cells include T-cells, smooth muscle cells and thrombocytes; therefore, some of the ^68^Ga-pentixafor-positive uptake sites might represent very early-stage lesions without markedly elevated inflammation [55].

In a study of oncology patients, increased uptake of ^68^Ga-pentixafor was associated with an increased incidence of CV risk factors [62]. In line with previous results [63], the authors demonstrate an inverse relationship between ^68^Ga-pentixafor and FDG uptake with the degree of calcification [55]. Non-calcified sites demonstrated the highest uptake, whereas severely calcified plaques presented with the lowest uptake for both tracers [55].

^68^Ga-pentixafor uptake was observed in 1411 sites in 51 patients and was significantly associated with calcified plaque burden and CV risk factors, including age, arterial hypertension, hypercholesterolemia, history of smoking, and prior cardiovascular events [63]. Increased uptake was observed in patients with a higher risk profile, and may serve to successfully identify individuals with vulnerable plaque [63].

While insightful into CV risk and identifying early lesion development, no definitive conclusions can be drawn about its exact cellular source. Does ^68^Ga-pentixafor uptake represent the sum of all CXCR4-expressing cells localised within or near a particular lesion? [57,59,61,64]. Furthermore, the comparison between ^68^Ga-pentixafor to FDG resulted in only a weak correlation between tracers. Further studies are highly warranted to elucidate the underlying biological mechanisms and sources of CXCR4 to improve understanding of the clinical utility of this radiotracer.

## 7. Experimental/Novel PET Imaging Radiotracers including Their Studies in Animals

Animal models have facilitated the understanding of underlying mechanisms contributing to atherosclerotic plaque stability and monitoring disease progression. Moreover, using mice, or other animals for that matter, can be used for proof-of-concept studies, or to assess radiotracer behaviours in vivo. Furthermore, ex vivo validation of readings can be confirmed via gamma-counting, autoradiography, and immunohistochemistry for improved quantification [65]. Here, we will discuss novel radiotracers targeting hypoxia, matrix metalloproteinases, macrophage markers and various cell surface markers.

### 7.1. Hypoxia

Hypoxia has been reported in plaques from humans and animal models of atherosclerosis. In atherosclerosis and vascular disease of larger arteries, hypoxia occurs within layers of the arterial wall [66]. Hypoxia stimulates pro-atherosclerotic processes, including deficient lipid efflux, inflammation, interference with macrophage polarization, and glucose metabolism [67]. The exact mechanisms of hypoxia in atherosclerosis remain unclear, but may involve hypoxia-inducible factor (HIF)-1α and NF-κB, signalling pathways implicated in inflammation and hypoxia [66]. Both HIF-1α and NF-κB are activated by the same pro-inflammatory stimuli (TNF-α and IL-6), disturbed blood flow and oxidative stress [68,69].

In concert with inflammation, hypoxia also triggers metabolic glucose changes to maintain ATP production in cells. Under these conditions, HIF-1α triggers glycolytic gene activation in endothelial cells, giving rise to enhanced cell proliferation and inflammation [69,70]. HIF-1α also activates endothelial to mesenchymal cell transition, further enhancing inflammation, proliferation and permeability [71,72,73,74]. Altogether these changes in endothelial cell function are a hallmark of a dysfunctional endothelium that leads to the development and progression of atherosclerosis.

In a rabbit model of atherosclerosis, ^18^F-fluoromisonadazole uptake has been demonstrated. Uptake is increased and correlates with advanced disease progression and aligns in regions rich with macrophage population and neovascularization [75] (Figure 1).

Altogether, there is substantial evidence that there are regions within the plaque in which significant hypoxia exists that may change the function, metabolism and responses of many cell types found within the developing plaque, and dictate whether the plaque will evolve into a stable or unstable phenotype [66].

### 7.2. MMP and Degradation

Matrix metalloproteinases (MMPs) play a key role throughout all stages of atherosclerosis and are involved in vascular inflammation, smooth muscle cell migration, endothelial dysfunction, extracellular matrix degradation, vascular calcification, and plaque activation and destabilization [76]. They are secreted by a range of cells including macrophages, neutrophils, lymphocytes, endothelial cells, vascular smooth muscle, fibroblasts, and osteoblasts [76].

Previous studies examining the role of MMP associate increased expression with morphological changes in diseased arteries of experimental models of atherosclerosis [77]. Increased amounts of MMP-7 and -9 have been observed in unstable plaques, with the highest expression of MMP-9 observed in plaques of lipid types compared to those of necrotic and inflammatory-erosive types [76]. MMP-9 correlates positively with the size of the necrotic core of coronary atherosclerotic plaques in stable CAD patients [78]. Serum levels of MMP-9 and the MMP-9/TIMP-1 ratio may be valuable in ACS diagnoses and prognosis, with MMP-9 activation in serum associated with poor CV outcome [78,79]. Moreover, elevated serum MMP-9 concentration has been independently associated with a high total carotid artery plaque score, plaque instability, and large intima media thickness value [78,80].

In models of atherosclerosis, ^123^I- or ^125^I-labelled CGS 27023A, a broad spectrum MMP inhibitor [81], and RP-805, a ^99m^TC-labelled broad-spectrum MMP-inhibiting macrocyclic compound, have been shown to bind to atherosclerotic plaque and show early promise in translatable MMP imaging [82,83] (Figure 1).

### 7.3. Activated Macrophages via Mannose Receptor

The mannose receptor is over-expressed in activated macrophages. Authors utilising the novel ^111^In-tilmanocept radiotracer observed in vivo and ex vivo (autoradiography) uptake in atherosclerotic plaques of *ApoE-/-* mice. The study also observed ^111^In-tilmanocept accumulation in macrophage rich organs [84] (Figure 1).

Another novel mannose receptor-targeting radiotracer, ^68^Ga-NOTA-MSA (neomannosylated human serum albumin), demonstrated ex vivo binding capability to peritoneal murine macrophages and in the aorta of atherosclerotic rabbit models [85] (Figure 1). The uptake of ^68^Ga-NOTA-MSA PET/CT was higher in atherosclerotic animals compared to control and were not different from ^18^F-FDG-PET/CT imaging.

### 7.4. Chemokine Receptor Targeting in Atherosclerosis

Chemokine receptors are involved throughout the process of atherosclerosis, including roles in plaque initiation, progression, destabilization, and rupture via leukocyte recruitment and inflammation.

The broad-spectrum chemokine receptor antagonist ^64^Cu-vMIP-II-Comb uptake increased in line with plaque progression in the mouse model of atherosclerosis [86]. Uptake correlated with enlarged plaque, increased macrophage population and elevated chemokine receptor expression. ^64^Cu-vMIP-II-Comb uptake was confirmed by reverse transcription polymerase chain reaction of chemokine receptors and histopathological characterization of plaque. The study demonstrates the potential to use ^64^Cu-vMIP-II-Comb to determine plaque progression.

### 7.5. Macrophage Scavenger Receptor (SR-A1)

SR-A1 is expressed by macrophages in the cap area, inside the lesion, but not by vascular smooth muscle cells or endothelial cells in non-plaque areas. Additionally, SR-A1 exacerbates atherosclerosis by promoting foam cell formation and secretion of pro-inflammatory cytokines.

Uptake of ^89^Zr-Mal-HAS in atherosclerotic lesions of *ApoE-/-* mice was higher compared to ^18^F-FDG, and the difference compared to wild-type mice indicates increased specificity for macrophage-targeted imaging, especially in early atherosclerosis [87]. ^89^Zr-Mal-HSA appears to be a promising diagnostic tool for the early identification of macrophage-rich areas of inflammation in atherosclerosis.

Altogether, the plaque microenvironment is highly dynamic and complex. The wide range of pathophysiological pathways that contribute to the disease pathology enable radiotracer flexibility to exploit targets for imaging. 

### 7.6. Challenges in Animal PET Imaging

The most studied models of atherosclerosis are the *ApoE-/-* and *LDLR-/-* murine models. They are well established and have been highly characterised. However, when it comes to imaging these mice, the challenge becomes apparent. High spatial resolution is crucial in murine plaques. The largest murine plaques are located in the aorta, which has a diameter of ~1 mm [65]. This makes the plaques themselves small and contain relatively few target cells, which can affect receptor expression depending on the target.

## 8. *ApoE-/-* Mouse PET Imaging

In our study, twenty-seven 20-week-old male *ApoE-/-* mice were fed a high fat diet for 12 weeks and injected with ^18^F-FDG (*n* = 21), ^18^F-NaF (*n* = 3), or ^68^Ga-DOTATATE (*n* = 3) radiotracer (Appendix A). All mice underwent whole-body PET CT using InVivoScope software (Bioscan Inc, California, U.S.A) at approximately one-hour post-administration of the radiopharmaceutical. Low-dose CT was performed for attenuation correction and anatomical localisation. Mice were culled after the completion of one-hour uptake time to reduce motion artifacts from mouse orientation and movements and to overall improve the image quality and semi-quantitation.

All animal experiments and procedures were approved by the local ethics committee (Harry Perkins Institute of Medical Research (AE114) and the University of Western Australia (F71731).

**Figure 2:**
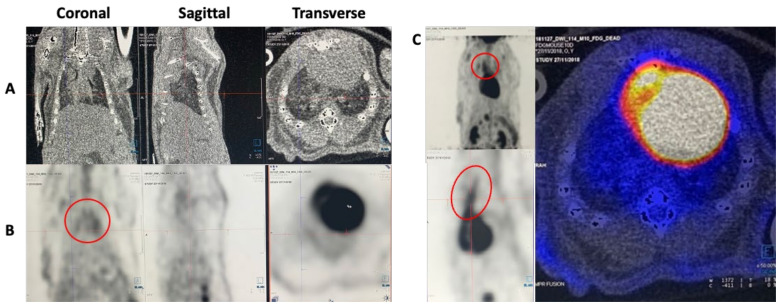
^18^F-FDG-PET scan of a 20-week-old male *ApoE-/-* mouse. (**A**) Representative CT images for anatomical localisation. (**B**) Representative PET images showing ^18^F-FDG uptake in the aortic arch (circled). (**C**) Representative PET images (coronal, sagittal) showing ^18^F-FDG uptake in the ascending aorta indicated by the circled areas. Representative fused PET/CT image with increased ^18^F-FDG uptake in the aortic arch (right panel). ^18^F-FDG: ^18^F-fluorodeoxyglucose, PET: Positron Emission Tomography, CT: Computed tomography.

Using Syngio.via VB40 software (Siemens Healthineers, Bayswater, Australia), we assessed the efficacy of these three imaging agents in plaque identification in the *ApoE-/-* murine model. In mice given ^18^F-FDG, we observed ^18^F-FDG uptake in the ascending and arch of the aorta, where the plaque burden was expected to be high (Figure 2). In our experience, overnight fasting of the mice before imaging had not proven to be beneficial. Though all efforts were made to follow the animal protocol of the dietary preparation prior to the imaging (fasting from food for 4–6 h (water available)) to suppress myocardial activity, all mice demonstrated intense myocardial activity limiting the assessment of smaller calibre coronary vessels; however, the larger vessels such as the thoracic and abdominal aorta could be readily appreciated. We observed homogenous uptake in the aortic arch aside from three mice that demonstrated heterogeneous uptake like what we expect in patients who present with vulnerable active plaque in real time. It is feasible that the distribution noted was homogenous in cases where neighbouring plaque may have merged to form a uniform density.

**Figure 3:**
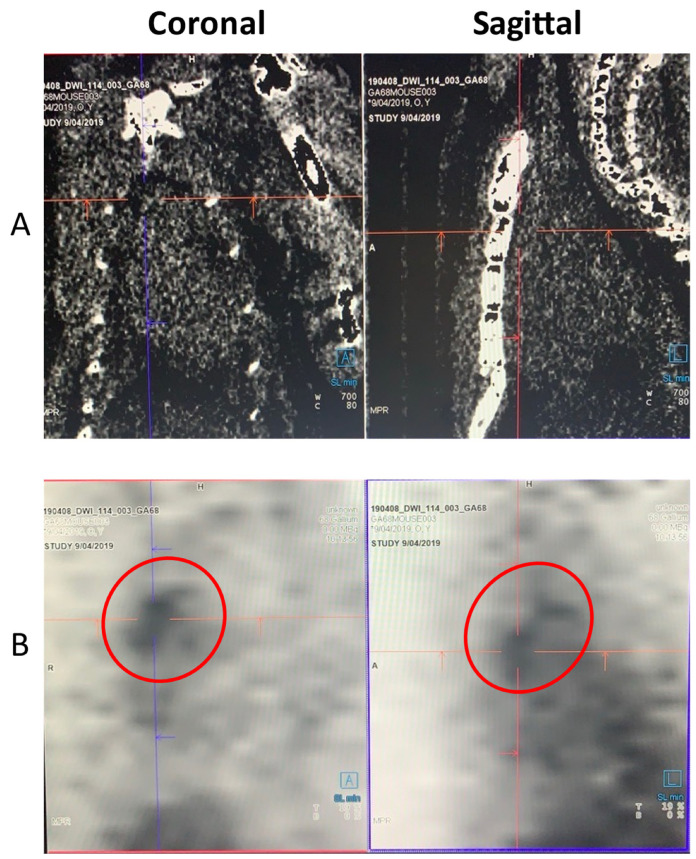
^68^Ga-DOTATATE PET scan of a 20-week-old male *ApoE-/-* mouse. (**A**) Representative CT images for anatomical localization. (**B**) Representative PET images of 68Ga-DOTATATE uptake in the ascending aorta indicated by the circled areas. 68Ga-DOTATATE: Gallium DOTATATE, PET: Positron Emission Tomography, CT: Computed tomography.

Unlike ^18^F-FDG, which exhibited intense homogenous myocardial uptake, we observed no background abnormal myocardial binding in mice given ^68^Ga-DOTATATE. Only one mouse showed low-grade uptake in the ascending aorta in agreement with the ^18^F-FDG PET CT (Figure 3). We found the ^68^Ga-DOTATATE images were noisier compared to the other radiotracers, likely from increased radiotracer decay at the time of scanning due to the ^68^Ga short t_1/2_ (68 min), resulting in lower-than-expected whole body tracer concentration further jeopardising the image quality. The physical characteristics of ^68^Ga also likely contribute to the poor image quality due to the longer positron range and higher positron energy [88]. This makes ^68^Ga less ideal for imaging, especially in mice as they can limit special resolution [88].

^18^F-NaF-imaged mice produced excellent skeletal images; however, they did not identify any plaque calcification (image not shown). This was likely due to the mice being too young, or the imaging being performed too early in the disease course to observe changes from atherosclerosis. ^18^F-NaF by far produced the cleanest quality images out of all three radiopharmaceuticals.

## 9. General Considerations in PET Imaging

Unfortunately, while PET is widely flexible and applicable to many disease diagnoses, PET imaging has a number of limitations. With a limited spatial resolution of 3–5 mm, making reproducible measurements in the right coronary artery and mid to distal vessels becomes problematic. The inability to optimally suppress glucose uptake by the myocardium can interfere with ^18^F-FDG visualisation and quantification in coronary plaques. Furthermore, image clarity is of concern due to cardiac and respiratory motion. In efforts to mitigate these issues, some investigators perform end-diastolic imaging using only part of the PET scan at the cost of increased image noise. Additionally, PET images require co-registration with CT or MRI for localisation, which are both hindered by cardiac and respiratory motion [89].

Another challenge of PET imaging is the availability of radiotracers. Although many radiotracers have been proven to be useful for identifying atherosclerotic plaque in preclinical models of disease, the majority of the clinical studies imaging coronary plaque have been performed using either ^18^F-FDG or ^18^F-NaF. This is in part due to the ease of tracer synthesis, their availability, and their history in clinical applications.

The expanding number of tracers available that can target receptors expressed by macrophages, luminal endothelial cells, hypoxia, and angiogenesis have been used to detect active atherosclerotic plaque, but only in small clinical cohorts. The successful translation of these tracers to clinical practice will depend on availability, affordability and ease of synthesis.

In addition, confirmation of plaque vulnerability needs to be correlated with increased plaque tracer uptake after the atherothrombotic event has occurred. To demonstrate the association, it will be necessary to show that increased tracer uptake by the coronary artery plaque is associated with a future cardiac event.

Finally, to really enable feasible clinical molecular imaging, major advances in image acquisition and processing are required to address limitations imposed by cardiac and respiratory motion when assessing plaque.

## 10. Future Direction and Imaging Strategies

The scope of imaging inflammation needs to increase to incorporate more inflammatory pathways identified by basic science. The understanding of particular inflammatory cytokines and their role in atherogenesis provides additional opportunity for radiotracer development.

Altogether, enhanced inflammation has been identified as a marker of the risk of post-infarct ventricular dysfunction and heart failure [90]. Overactive and/or prolonged inflammation (both myocardial and vascular) during ACS can contribute significantly to cardiac damage and dysfunction and adverse clinical outcomes [90]. Patients experiencing recurrent events have a more active innate immune system, with clinical data suggesting that acute myocardial infarction can accelerate plaque development by expanding the systemic leukocyte pool [91]. In patients with stable angina, the presence and extent of myocardial infarction has been associated with increased aortic atherosclerotic inflammation via increased FDG uptake and early recurrent myocardial infarction [92].

The study of C-reactive protein (CRP), interleukin (IL)-1β, and NLR family pyrin domain-containing 3 (NLRP3) inflammasome has revealed critical roles for each in post-infarction systemic inflammation and progression of atherosclerosis [92,93,94,95,96,97,98,99]. Higher levels of NLRP3 in ACS patients rather than CAD patients indicate that it is not only elevated in chronic atherosclerosis, but also in the acute phase of the atherosclerotic process [93]. Moreover, activation of NLRP3 during myocardial infarction in cells other than myocytes (including endothelial cells, neutrophils, and fibroblasts) has been shown to contribute indirectly to cardiac dysfunction [94]. Downstream cytokines IL-1β (also shown to affect cardiac dysfunction [100]) and IL-18 were also elevated in these ACS patients [93]. IL-1β and NLRP3 have been positively correlated with the extent of coronary atherosclerosis as assessed by SYNTAX score and CLINICAL SYNTAX score, while NLRP3 has also been positively correlated with the GENSINI score and lesion characteristics of coronary syndrome patients [93].

Therapeutically, administration of IL-1β-inhibiting agents (canakinumab, rilnacept, anakinra) result in a significantly lower rate of recurrent cardiovascular events and prevent future hospitalisation for heart failure [100]. Moreover, canakinumab has been shown to inhibit systemic inflammatory response post-myocardial infarction and reduce new-onset heart failure and hospitalisation [98].

Building on these strong correlations, utilising baseline systemic NLRP3 concentration is a promising prognostic utility and, through correlation with GRACE and TIMI risk scores, could prove an efficient event predictor for MACE [93]. Developing radiotracers specific to NLRP3 and associated inflammatory proteins (IL-1β and IL-18) would improve cardiac event prediction and improve timing of intervention. With the success of these therapeutic interventions, developing PET radiotracers to target NLRP3-associated proteins would greatly improve patient outcome and treatment opportunity. Together, targeting vulnerable plaques and post-ACS imaging would facilitate greatly enhanced patient treatment and outcomes.

## 11. Clinical Implications and Conclusions

Despite progress in understanding the complex underlying biology of atherosclerosis, it remains a global health problem. Improved knowledge of the disease mechanisms has translated to an increase in novel radiotracer development and leaps forward in plaque characterisation imaging. However, to prospectively recognise vulnerable plaque and prevent the occurrence of adverse events, an imaging strategy that targets the molecular changes in vulnerable plaque is needed. While biomarkers assess systemic inflammation, a plaque marker of susceptible rupture would be invaluable in the process of targeted local intervention and primary prevention of adverse events. Moreover, including inflammatory pathways in therapeutic targets could bring PET imaging diagnosis and intervention closer together to effectively target cardiovascular outcomes and improve patient outcome.

## Appendix A. Preclinical PET/CT ^18^F-FDG Imaging Protocol

Animal preparation included fasting from food for 4–6 h (water available). The animals were warmed for 30 min (at approximately 30 °C) prior to administration of ^18^F-FDG. Anaesthesia with isoflurane was administered, followed by intravenous (IV) or intraperitoneal (IP) injection of ~20 MBq of ^18^F-FDG, in a volume no greater than 200 μL for IV injection. An uptake phase of 60 min (with the animal kept warm) under anaesthesia was followed by ex vivo animal PET- CT scan on the Bioscan BioPET/CT 105 camera. Proprietary and/or InVivoScope software was used for image analysis. The total time under anaesthesia was about 90 min.

Preclinical PET/CT ^18^F-NaF & ^68^Ga Imaging Protocol:

Animal preparation included fasting from food for 4–6 h (water available). The mice were warmed for 30 min (at approximately 30 °C) prior to administration of radiotracer. Anaesthesia with isoflurane was administered, followed by IV injection of ^18^F-NaF (~18 MBq) or IP injection of ^68^Ga -DOTATATE (~10 MBq), in a volume no greater than 200 μL for injection. An uptake phase of 60 min (with animal kept warm) under anaesthesia was followed by ex vivo animal PET-CT scan on the Bioscan BioPET/CT 105 camera. Proprietary and/or InVivoScope software (California, U.S.A.) was used for image analysis. The total time under anaesthesia was about 40 min.
